# Genetic Diversity and Host Alternation of the Egg Parasitoid *Ooencyrtus pityocampae* between the Pine Processionary Moth and the Caper Bug

**DOI:** 10.1371/journal.pone.0122788

**Published:** 2015-04-09

**Authors:** Shahar Samra, Murad Ghanim, Alex Protasov, Manuela Branco, Zvi Mendel

**Affiliations:** 1 Department of Entomology, Volcani Center, Bet Dagan, Israel; 2 Faculty of Agriculture, the Hebrew University of Jerusalem, Rehovot, Israel; 3 Centro de Estudos Florestais (CEF), Instituto Superior de Agronomia, Technical University of Lisbon, Lisbon, Portugal; Institute of Vegetables and Flowers, Chinese Academy of Agricultural Science, CHINA

## Abstract

The increased use of molecular tools for species identification in recent decades revealed that each of many apparently generalist parasitoids are actually a complex of morphologically similar congeners, most of which have a rather narrow host range. *Ooencyrtus pityocampae* (OP), an important egg parasitoid of the pine processionary moth (PPM), is considered a generalist parasitoid. OP emerges from PPM eggs after winter hibernation, mainly in spring and early summer, long before the eggs of the next PPM generation occurs. The occurrence of OP in eggs of the variegated caper bug (CB) *Stenozygum coloratum* in spring and summer suggests that OP populations alternate seasonally between PPM and CB. However, the identity of OP population on CB eggs seemed uncertain; unlike OP-PPM populations, the former displayed apparently high male/female ratios and lack of attraction to the PPM sex pheromone. We studied the molecular identities of the two populations since the morphological identification of the genus *Ooencyrtus*, and OP in particular, is difficult. Sequencing of COI and ITS2 DNA fragments and AFLP analysis of individuals from both hosts revealed no apparent differences between the OP-PPM and the OP-CB populations for both the Israeli and the Turkish OPs, which therefore supported the possibility of host alternation. Sequencing data extended our knowledge of the genetic structure of OP populations in the Mediterranean area, and revealed clear separation between East and West Mediterranean populations. The overall level of genetic diversity was rather small, with the Israeli population much less diverse than all others; possible explanations for this finding are discussed. The findings support the possibility of utilizing the CB and other hosts for enhancing biological control of the PPM.

## Introduction

The term "host alternation" is often used to describe situations in which species are required to change hosts constantly in order to survive, as in the case of true parasites that exhibit complex life cycles that require two different host species [1], or in the case of aphid species that alternate seasonally between two distinct host plant species [2]. Host alternation by parasitoids is rarely described, although the term is sometimes used to describe a more general phenomenon of switching between different host species [3]. In most known cases of parasitoids having multiple host species, the changing of hosts does not necessarily occur on a regular or mandatory basis, and the term "host switch" (or "switching") is used more often [4,5].

Although most parasitoid species are assumed to have rather narrow host ranges, some apparently cover wide ranges and are able to switch regularly between different host species [6–9]. However, wide application of molecular techniques in recent decades revealed that many of those designated as "generalist parasitoids" are actually a complex of several closely related and morphologically similar species each [10,11], also described as "cryptic" or "sibling species" [12,13]. These segregated species often utilize different hosts; many of them have rather narrow host ranges and can be considered specialists [10,11]. Species divergence which occurred on the basis of differences in host utilization is described in the literature as "Host Associated Differentiation" [14,15]. In cases where morphological differences between closely related parasitoid species are sparse or cannot be detected, the distinction can be revealed through molecular analysis of genetic distances [12,16]. *Ooencyrtus pityocampae* Mercet (OP) (Hymenoptera: Encyrtidae) is a well-studied egg parasitoid of the pine processionary moth (PPM) *Thaumetopoea pityocampa* (Den and Schiff)/*T*. *wilkinsoni* Tams species complex (Lepidoptera: Notodontidae). The moth is a major defoliator of pines throughout the Mediterranean basin [17–20]. OP is widely distributed within the moth's distribution range and is considered one of its most important mortality agents. However, parasitism rates are highly variable among and within regions [21–30]. OP is common in Israeli pine forests, where it is the dominant egg parasitoid attacking the PPM eggs in this area, responsible for substantial egg mortality rates—up to 80% in a single egg mass [21].

The PPM egg masses are deposited on pine needles in late summer and autumn, i.e., from late August to November, depending on the location, and can support two or three generations of OP. Individuals mainly of the last generation undergo winter diapause inside host eggs, as last-instar larvae [21,25,29,31,32]. However, most diapausing OP individuals emerge in spring and early summer—mainly from April through June—of the following year, 2–4 months prior to the appearance of the first PPM egg masses. This asynchrony between the periods of PPM oviposition and OP emergence was observed in several areas around the Mediterranean [22,25,31–33]. OP adults may survive for up to about 100 days under laboratory conditions [32], therefore some individuals might survive until the first PPM eggs appear. However, this scenario seemed unlikely with such a long time gap. Another possibility is that OP exploits other hosts during the spring and summer, when PPM eggs are absent. In fact, OP is often considered a generalist parasitoid, as it is known to attack the eggs of several species of Lepidoptera and Heteroptera [34–38]. However, until recently this wasp was never found on alternative hosts in spring and summer, outside the autumnal main activity period of the PPM, but this situation changed when, about 10 years ago, it was discovered that OP reproduces on the eggs of the variegated caper bug (CB) *Stenozygum coloratum* (Hemiptera: Pentatomidae), which is found on caper plants growing within and on the edges of pine forests in Israel [32]. The CB is found in the Middle East and East Africa [39,40]; its oviposition period lasts throughout the spring and summer, mainly from May through September [39], and egg parasitism by OP occurs throughout this period [32]. Recently, OP was also found in a unique allochronic summer PPM population, discovered in Leiria, Portugal. This population reproduces in spring and early summer, and its eggs are available for OP parasitism as early as late April and early May [41]. As far as we know, the CB and summer PPM population are the only reported cases of the parasitoid being found parasitizing alternative hosts outside the PPM oviposition period. OP was also supposedly found on the eggs of *Brachynema signatum* (Hemiptera: Pentatomidae), a pest of pistachio orchards in Iran [36], although the high male/female ratio in this population, is atypical of OP populations [21], indicating that other *Ooencyrtus* species were present, therefore this report should be further validated.

**Fig 1:**
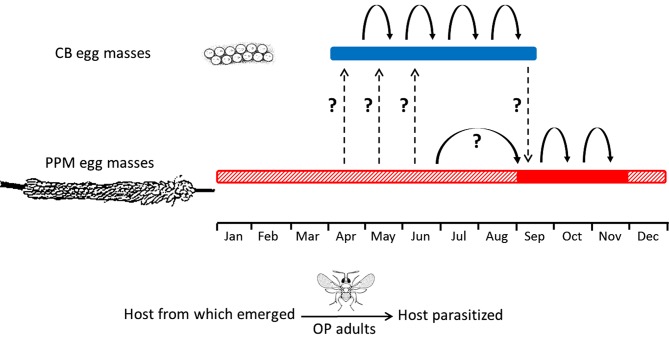
Schematic illustration of seasonal activity pattern of *Ooencyrtus pityocampae* (OP) and availability of its known hosts in Israel. Red bar represents the occurrence of egg masses of the pine processionary moth (PPM) *Thaumetopoea wilkinsoni*; vital (solid line) or after larval hatching (dashed line). Blue bar represents the occurrence of egg masses of the caper bug (CB) *Stenozygum coloratum*. Dashed arrows indicate OP activity (hosts from which adults emerged and parasitized hosts: arrow tail and head, respectively); solid arrows indicate parasitism of the same host species. Dashed arrows indicate occurrence of host alternation. Question marks indicate unconfirmed data. Data is based on Mizrachi [32].

In Israel, the CB stops reproducing around the beginning of autumn, i.e., September-October [39], which corresponds more or less to the beginning of PPM oviposition period [42]. Hence, we hypothesized that OP populations alternate seasonally between these two hosts (Fig 1). In light of this background it was further suggested that CB might be an important element in the conservation of OP during spring and summer. Therefore, from an applicative point of view, increasing CB population could potentially improve biological control of PPM.

Nevertheless, doubts concerning the identity of the parasitoid population on the CB eggs had emerged, which raised questions regarding the host alternation hypothesis; in general, several different factors could prevent a parasitoid from being able to exploit a specific host [43]. Even in the absence of a physiological barrier, parasitism can be impeded by ecological or behavioral constraints, such as differing activity periods, or a lack of attraction of the parasitoid to the host habitat, host plants, or host cues [44,45]. Consequently, many parasitoid species exploit only a fraction of the range of physiologically suitable host species [43].

The ability of OP individuals that parasitize PPM and CB to develop in and switch successfully between these two hosts was confirmed in laboratory trials [32], which indicates that both hosts are physiologically suitable for development of the two OP populations. However, field studies have shown that unlike the autumn OP population on the PPM, the summer OP population on the CB does not seem to be attracted to the PPM sex pheromone [32]. Furthermore, the parasitoid population on the CB apparently contained a relatively high proportion of males: about 25% [32] as compared with 0–2.6% on the PPM populations in Israel [21] and other areas [24,25,30,31,46]. OP is known as a uniparental species (the females are parthenogenetic), and the high male/female ratio in the CB population remained unexplained until recently, when it was affirmed that these males belong to other *Ooencyrtus* spp. (These data is about to be published separately). Furthermore, the identification of individuals emerging from PPM and CB eggs, as well as from other known hosts, was based on morphological characteristics, which may be unreliable in this species (J. Noyes and E. Guerrieri 2014, personal communication). As a result, doubts concerning the identity of the parasitoid population on the CB eggs were raised.

The question was whether the "two" OP populations were in fact a single one whose individuals alternated seasonally between the PPM and CB, or whether these individuals represented two distinct populations (or separated species), each specializing on a different host. Our preliminary study, which compared COI sequences of OP individuals from both host species in different areas in Israel, revealed the presence of five closely related, morphologically similar *Ooencyrtus* spp in the CB eggs (The presence of other species was later confirmed by three taxonomic specialists, G. Japoshvili, J. Noyes and E. Guerrieri); one of the congeners was genetically identical to OP populations occurring in PPM eggs. These findings supported the alternation hypothesis, and accounted for the high male/female ratio of the CB parasitoid population, because the males were shown to belong to the other *Ooencyrtus* spp. [47].

However, the overall level of genetic differences between OP individuals was extremely low, practically zero, according to the DNA fragment used—therefore it could not be determined whether the lack of genetic differences between the two populations obtained from the two respective host species was because they were a single population, or whether the resolution of the genetic analysis was not high enough to detect differences between closely related populations, possibly because the chosen DNA segment was too conserved. This apparent lack of genetic diversity also raised the question of whether the low polymorphism found is unique to the Israeli OP populations, or is a general phenomenon in this species. To answer these questions three different approaches were taken. First, we extended our sample size and sampling area to include other regions in the Mediterranean basin, focusing on areas where both hosts are found, i.e., Israel and southern Turkey. Secondly, another DNA fragment (ITS2) was added to the analysis. Finally, a different genetic analysis, i.e., Amplified Fragment Length Polymorphism—AFLP [48] was also used to study the Israeli populations, in which no genetic diversity could be detected by sequencing analysis. The advantage of AFLP lies in the fact that genetic data from many different areas in the insect's genome are analyzed and compared, increasing the chances of recognizing differences in closely related populations. The AFLP method was used previously by Simonato [49] to study OP populations in Italy, and it revealed substantial polymorphism levels which enabled distinction between geographically close populations.

Thus, the objectives of the present study were as follows: (1) to confirm the presence or absence of host alternation in OP, by determining the level of genetic similarity between OP populations from PPM and CB; and (2) to determine whether the apparently low genetic diversity in the Israeli OP population is unique to this area, or is a general phenomenon in this species. To address these issues, data on the genetic structure of several OP populations from several locations around the Mediterranean basin were obtained and analyzed.

## Materials and Methods

### Sampling

During 2010–2013 egg masses of PPM and CB were collected from the field in six countries, four in the East Mediterranean region, i.e., Israel, Turkey, Greece and Cyprus, and two in the West Mediterranean region, i.e., Portugal and Tunisia. No specific permissions were required for collecting all the samples in this study in all locations presented in Table 1, and this study did not involve endangered or protected species. Israel and Turkey were the most extensively sampled, because populations of both hosts, PPM and CB, occur in both countries. In Israel, sampling was conducted in or near planted pine forests in the northern part of the country, i.e., the Galilee Mountains and Valleys—referred to as "Israel-north"—and in the southern Judean Hills—"Israel-south". In Turkey, the Hatay region and Mersin-Adana districts were sampled; these areas were chosen for their large pine forests, where both PPM and CB are relatively common. CB egg masses were collected from *Capparis* spp., and PPM egg masses from the various pine species that were present on each site. Each egg mass was placed in a separate glass tube sealed with cotton wool. The emerging OP individuals were collected in Eppendorf tubes filled with 96% ethanol and stored at -20°C. It was attempted to obtain individuals from both hosts and in each sampling area, but this was not always possible, for several reasons. First, the CB is found in the East Mediterranean area, and its distribution only partially overlaps that of the PPM [47], and even where both hosts are known to occur, e.g., in Turkey, Cyprus and Israel, CB eggs could not always be found. Secondly, in every location where the CB egg masses were collected, other *Ooencyrtus* spp. were also parasitizing the eggs, usually substantially outnumbering OP; in some locations no OP individuals were found in the CB eggs sampled, e.g., the Negev desert and Jordan Valley (see discussion), therefore these samples were not included in the present work.

**Table 1:** Information on *Ooencyrtus pityocampae* samples used in the present study.

**Locality code**	**Country** *	**Region/ district**	**Location**	**Co-ordinates**	**Altitude (m)**	**Collection date**	**Host**	**# ind.**	**Haplotype Composition**
1	Israel^1^	Southern Judean Hills	Yatir	31°20'N; 35°05'E	640	22-Nov-10	PPM	3	H_1(3)
2a	Israel^1^	Southern Judean Hills	Lahav	31°22'N; 34°51'E	480	10-Jun-12	CB	15	H_1(15)
2b	Israel^1^	Southern Judean Hills	Lahav	31°22'N; 34°51'E	480	9-Nov-12	PPM	11	H_1(10), H_2(1)
3a	Israel^1^	Judean Foothills	Haruvit	31°45'N; 34°55'E	135	4-Jun-11	CB	14	H_1(14)
3b	Israel^1^	Judean Foothills	Haruvit	31°45'N; 34°55'E	135	4-Nov-10	PPM	10	H_1(10)
4	Israel^1^	Judean Foothills	Eshtaol	31°48'N; 35°00'E	250	26-Jul-11	CB	3	H_1(3)
5a	Israel^1^	Lower Galilee	Gilboa	32°31'N; 35°22'E	200	4-Aug-10	CB	20	H_1(20)
5b	Israel^1^	Lower Galilee	Gilboa	32°31'N; 35°22'E	200	25-Oct-10	PPM	12	H_1(12)
6	Israel^1^	Western Galilee	Segev	32°52'N; 35°14'E	270	20-Oct-10	PPM	7	H_1(7)
7	Israel^2^	Upper Galilee	Rosh-Pinna	32°57'N; 35°32'E	380	12-Aug-11	CB	3	H_1(3)
8	Israel^1^	Upper Galilee	Biriya	33°00'N; 35°29'E	780	13-Oct-10	PPM	13	H_1(12), H_3(1)
9	Israel^1^	Upper Galilee	Naftali	33°13'N; 35°33'E	380	13-Oct-10	PPM	19	H_1(19)
10	Turkey^1^	Hatay	Antakya	36°12'N; 36°10'E	140	27-Jun-13	CB	36	H_1(4), H_3(21), H_4(11)
11	Turkey^3^	Hatay	Üçırmak-Altınözü	35°58'N; 36°11'E	500	23-Jan-11	PPM	34	H_1(1), H_3(2), H_5(4), H_7(27)
12	Turkey^4^	Adana	Karataş	36°33'N; 35°32'E	26	2-Jul-13	CB	18	H_3(15), H_ 6(3)
13	Turkey^3^	Mersin	Mersin	36°49'N; 34°34'E	15	18-Feb-11	PPM	6	H_3(6)
14	Turkey^3^	Mersin	Mezitli	36°46'N; 34°29'E	120	18-Feb-11	PPM	24	H_3(21), H_8(4)
15	Turkey^1^	Mersin	Tarsus	36°57'N; 34°54'E	50	28-Jul-13	CB	5	H_3(3), H_ 5(2)
16	Cyprus^5^	Limassol	Kellaki	34°48'N; 33°09'E	630	1-Oct-13	PPM	1	H_9(1)
17	Cyprus^5^	Paphos	Lysos	34°59'N; 32°30'E	540	2-Oct-13	PPM	6	H_6(1), H_7(2), H_10(3)
18	Greece^6^	Central Greece	Attiko Alsos, Athens	37°58′N; 23°43′E	290	1-Dec-12	PPM	5	H_7(4), H_11(1)
19a	Portugal^7^	Setúbal	Pinhal das Freiras	38°35'N; 09°08'W	40	15-Sep-10	PPM	9	H_12(3), H_13(6)
19b	Portugal^7^	Setúbal	Pinhal das Freiras	38°35'N; 09°08'W	40	1-Jun-13	PPM	3	H_12(2), H_13(1)
20	Tunisia^7^	Homónima	Tunis	36°48′N; 10°11′E	20	12-Sep-11	PPM	4	H_13(2), H_ 14(1), H_15(1)
21	Tunisia^7^	Zaghouan	Jabal Mansour	36°15'N; 09°41'E	540	15-Sep-12	PPM	4	H_14(3), H_16(1)

**Fig 2:**
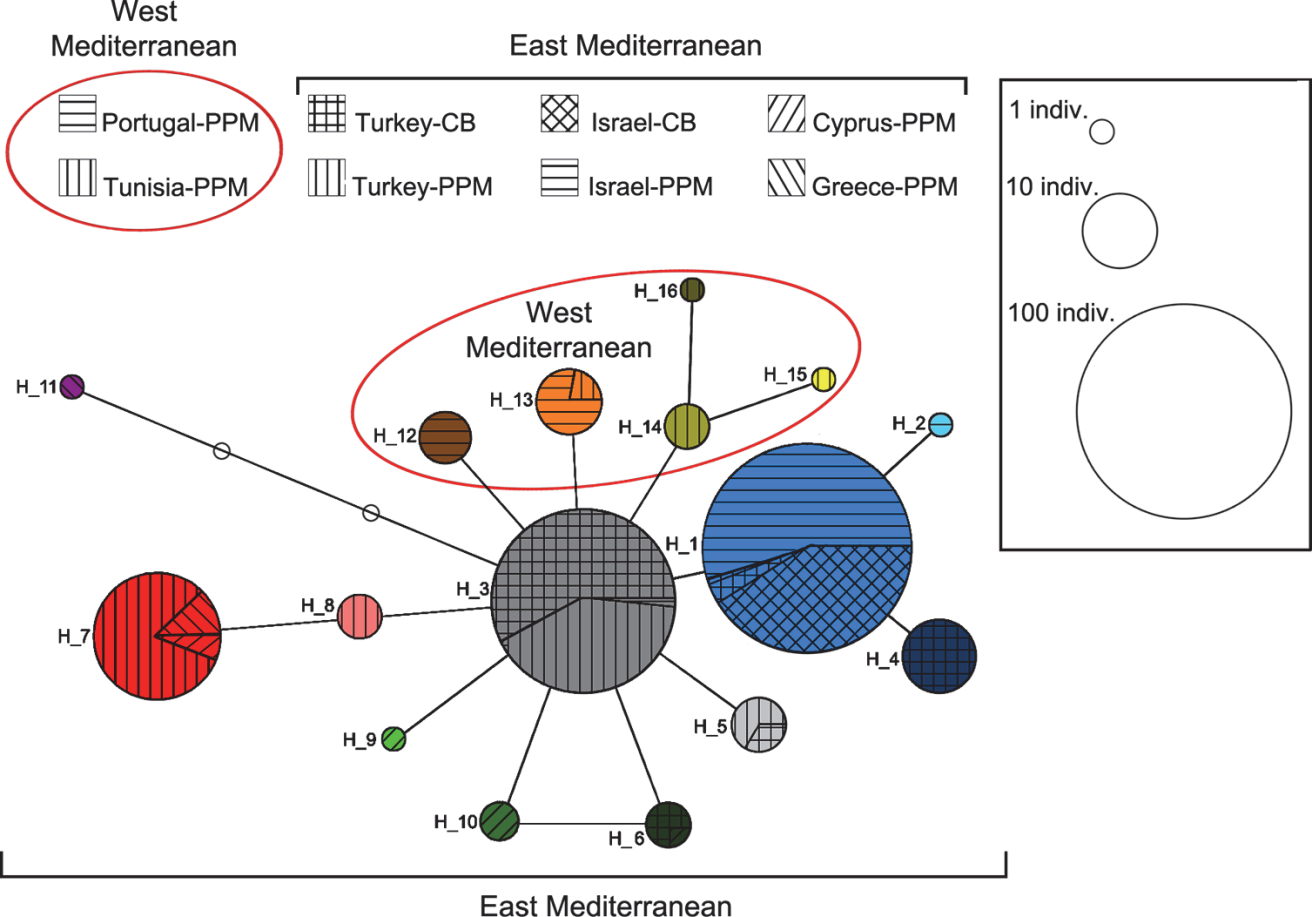
COI Haplotype network of *Ooencyrtus pityocampae*. The haplotype code is indicated next to each haplotype circle (corresponding to the codes in Table 1). Each haplotype was given a different color in order to enable the presentation of haplotypes' geographical distribution (see Fig 3). The size of each circle corresponds to the number of sampled individuals having this haplotype. Each line in the network represents a single mutational change. Empty circles indicate intermediate, missing haplotypes. Each country-host association in each of the main regions (East and West Mediterranean) is marked with a different line set (See legend above the network). Haplotypes belonging to the West Mediterranean populations (Portuguese and Tunisian) are encircled with red line.

Sampling sites, geographic co-ordinates, altitude, collection date, host species and haplotype composition for each locality. Haplotype codes are as in Fig 2. The number in parentheses after each haplotype code indicates the number of individuals with that haplotype. Locality codes refer to the sites shown in Fig 2. Codes for hosts are CB: Caper bug, *Stenozygum coloratum*; PPM: Pine processionary moth, i.e., *Thaumetopoea pityocampae* (Tunisia, Portugal and Greece) or *T*. *wilkinsoni* (Israel, Turkey and Cyprus).

* Collectors: 1. Shahar samra; 2. Carlos Jorge Carvalho; 3. Miktat Doğanlar; 4. Feza Can Cengiz; 5. Zvi Mendel 6. Panagiotis Milonas; 7. Manuela Branco.

In total, OP individuals were obtained from 21 locations, spread over six Mediterranean countries. In order to reduce the probability of sampling siblings, only a single individual wasp from each egg mass was used. A total of 285 OP individuals, comprising one to 36 individuals from each site/host, were used in the analysis. Among these, 114 emerged from CB eggs collected in Israel or Turkey, and 171 from PPM eggs collected in all six countries (see Table 1 for further data).

### Sequencing of COI and ITS2 gene fragments

#### DNA protocols

Total genomic DNA was extracted from each individual wasp, either with the Qiagen DNeasy Blood and Tissue kit (Qiagen, Redwood City, CA, USA) according to the manufacturer's protocol, or with Lysis buffer (40 μL reaction) made with the following reagents: 5 μL Tris 1M (pH 8), 1 μL 0.5M EDTA, 5 μL Igepal, 50 μL Proteinase-K (20 mg/mL) and 939 μL of double-distilled (dd) H_2_O, protocol as described in Chiel et al. [50].

Two DNA fragments were analyzed: a 946 bp fragment of the mitochondrial Cytochrom oxidase 1 (COI) gene, and a nuclear 931- to 933 bp fragment that comprised a partial (65 bp) fragment of the ribosomal 5.8S unit, a complete (523- to 525 bp) sequence of the ribosomal Internal Transcribed Spacer 2 (ITS2) and a partial (342 bp) fragment of the 28S ribosomal unit.

#### Primers and PCR conditions

The initial primer sequences used for COI were kindly provided by Marie-Anne Auger-Rozenberg, of INRA. They were:

forward—5' CGAATAAATAATATAAGTTTTTG 3' and

reverse—5' CAACATAAATAAGAATCTGGA 3').

However, the forward primer often attached to the middle of the COI segment and thus only the second half of the segment was obtained. To solve this problem, another forward primer was designed based on the previous one—5' CTCGAATAAATAATATAAGATTTTG 3'. The primers for ITS2 sequences were based on an alignment of ITS2 sequences of species of Chalcidoidea, which were available in NCBI. The forward and reverse primers were, respectively,

5' GAACTGCAGGACACATGAACA 3' and 5' CTTGTTCGCTATCGGTCTCGTGGT 3'.

Amplification of the COI and ITS2 used the following conditions: Initial denaturation at 95°C for 3 min, followed by 36 cycles of 95°C for 30 s, 54°C for 30 s, and 72°C for 75 s (COI) or 1 min (ITS2), followed by final extension at 72°C for 5 min.

The PCR for COI was performed in a 30-μL reaction volume containing a 3-μL sample, 0.3 μL (30 pmole) of each primer, 0.6 μL of dNTPs (10 mM of each), 0.15 μL (0.75 units) of Taq polymerase (Fermentas Ltd. Vilnius, Lithuania), 0.6 μL of MgCl_2_ (to a final concentration of 2.5 mM) and 22.05 μL of dd H_2_O. The PCR for ITS2 was performed with the same reagent concentrations except for the MgCl_2_, which was at a final concentration of 2 mM.

#### PCR product purification and sequencing

PCR products were electrophoresed through 1% Agarose gel, and the specific segment was cut from the gel and later extracted and purified with the RBC-YDF HiYield Gel/PCR DNA Fragments Extraction Kit (RBC, Minsheng Rd., Banqiao City, Taipei County 220, Taiwan). Sequencing was done by Macrogen Cor. (Seoul, Korea). The PCR products were sequenced with both forward and reverse primers, and the sequences were aligned by means of the ChromasPro software Version 1.6 (Technelysium Pty Ltd, South Brisbane, Queensland, Australia) and checked manually for errors.

#### Sequence data analysis

Since the sequencing of ITS2 did not reveal a significant level of polymorphism, the sequence analysis was based on the COI sequences alone. The COI sequences were aligned with Mafft (version 7) software [51] and translated into amino acid in order to verify that none of the sequences contained any stop codon, which could indicate the presence of numt. No gaps were present in the alignment.

The DNASP v5.10.1 program [52] was used to determine the haplotype and nucleotide diversity indices. A median-joining haplotype network [53] was reconstructed by using the NETWORK 4.2.0.1 program (available at http://www.fluxus-engineering.com), under default parameters.

Pairwise *Φ*_*st*_ values were computed by means of the Arlequin v.3.5.1.3 software [54], with 10,000 permutations, to investigate the genetic structure. Population differentiations were tested with an exact contingency test as implemented in Arlequin (with 1,000,000 Markov chain steps and 100,000 dememorization steps), and FDR correction [55] was applied to correct for multiple testing. To complement the information provided by *Φ*_*st*_ values, we also computed Jost’s [56] distance D, which measures allele-shared information between pairs of populations. Computations of pairwise D values and their confidence intervals were conducted with the SPADE program [57].

An analysis of molecular variance (AMOVA) was conducted with Arlequin, in order to determine the distribution of molecular variance among populations from the various studied areas. It was of particular interest to test for host related differences in the Turkish population, which unlike the Israeli population, showed relatively high level of diversity. A second AMOVA test was conducted to determine whether the genetic diversity was partitioned between West Mediterranean sites, i.e., Portugal and Tunisia, and East Mediterranean sites, i.e., Cyprus, Greece, Israel and Turkey. The sampled sites were partitioned either into "region", "host within region" and "within population" components, or into "host", "region for each host", and "within population" components. In Israel and Turkey the samples were partitioned into four subgroups based on locality, i.e., Hatay and Mersin-Adana regions (Turkey) and north and south (Israel), and according to their host, i.e., PPM or CB. The significance of the variance component was determined by using 10,000 permutations:

In order to determine whether genetic distances (*Φ*_*st*_ and D) were correlated with geographic distances, a Mantel test was performed, using zt [58] with 1,000,000 replications. Additionally, the Israeli and Turkish populations were subjected to Tajima's D neutrality test [59], to test for evidence for the occurrence of non-random selection processes, such as recent population expansion. Significance was tested with 1000 randomized simulations (also conducted with Arlequin).

### AFLP analysis of the Israeli OP population

The Israeli OP population, which showed almost no genetic diversity in the sequencing analysis, was further studied by using Amplified Fragment Length Polymorphism (AFLP) analysis [48], mainly to test the level of genetic similarity between the OP-PPM and OP-CB populations. The AFLP analysis was carried out by Keygene N.V. (Wageningen, the Netherlands). A total of 43 OP samples were fingerprinted (see Table 1 for specific sample data). The samples included: 41 individuals distributed between both hosts and between two sites in Israel (i.e., Lahav and Gilboa); one individual from Mersin, Turkey; and one from Pinhal das Freiras, Portugal. The latter two were used as outgroups. For location co-ordinates see Table 1. All samples were numbered and sent to Keygene without any data on sample content or origin.

Because the total amount of DNA from a single wasp was not sufficient for the analysis, clones of genetically similar individuals were used. Each sampled female (clone founder) was completely isolated from the presence of males, as a safety measure, and was allowed to parasitize 100 silk moth eggs. The daughters of each female were allowed to parasitize additional eggs, until about 30–50 wasps per clone were obtained, which usually required more than one generation. The DNA was extracted jointly from all offspring of each female founder with the Qiagen DNeasy Blood and Tissue kit (Qiagen, Redwood City, CA, USA) according to the manufacturer's protocol. One microgram of total genomic DNA of each clone was suspended in 50 μL of dd water and used for the analysis.

After screening of 48 primer combinations (PCs) (which included *Eco*RI/*Mse*I restriction enzyme sites), three PCs were selected based on fingerprints quality and polymorphism scores: E12/M55, E13/M50 and E13/M59 (E12 = 5’-(*Eco*RI)AC-3’, E13 = 5’-(*Eco*RI)AG-3’, M49 = 5’-(*Mse*l)CAG-3’, M50 = 5’-(*Mse*I) CAT-3’, M55 = 5’-(*MSe*I) CGA-3’ and M59 = 5’-(*Mse*I) CTA-3’). After generation of the fingerprints the samples were genotyped according to presence or absence of polymorphisms of AFLP markers. Size ranges of markers were 60–560 bp. The genetic information generated was subjected to three different genetic distance analysis procedures. The marker scores were used to carry out the genetic diversity analyses in order to categorize the samples according to their genetic similarity. The NTSYSpc software [60] was used to produce three similarity matrices consisting of similarity indices for all combinations of samples. The similarity matrices were calculated by using the Simple Matching (SM), Jaccard (Jc), and Dice (D) coefficients, respectively.

To visualize the relationships between the samples, dendrograms were generated by using SAHN (Sequential Agglomerative Hierarchical Nested) cluster analysis with the use of UPGMA (Unweighted Pair-Group Method, Arithmetic average) parameters for all three matrices. To evaluate to what extent the dendrograms were good representations of the similarity matrices, the cophenetic value matrix was calculated for each dendrogram and plotted against the original similarity matrix in order to obtain the cophenetic correlation, which indicates the degree to which the dendrograms represent the similarity matrices.

## Results

OP haplotypes found in the present study have been deposited in GenBank (accession numbers KM485909 to KM485924 (COI) and KM527071 to KM527091 (ITS2)).

### COI sequencing analysis

**Fig 3:**
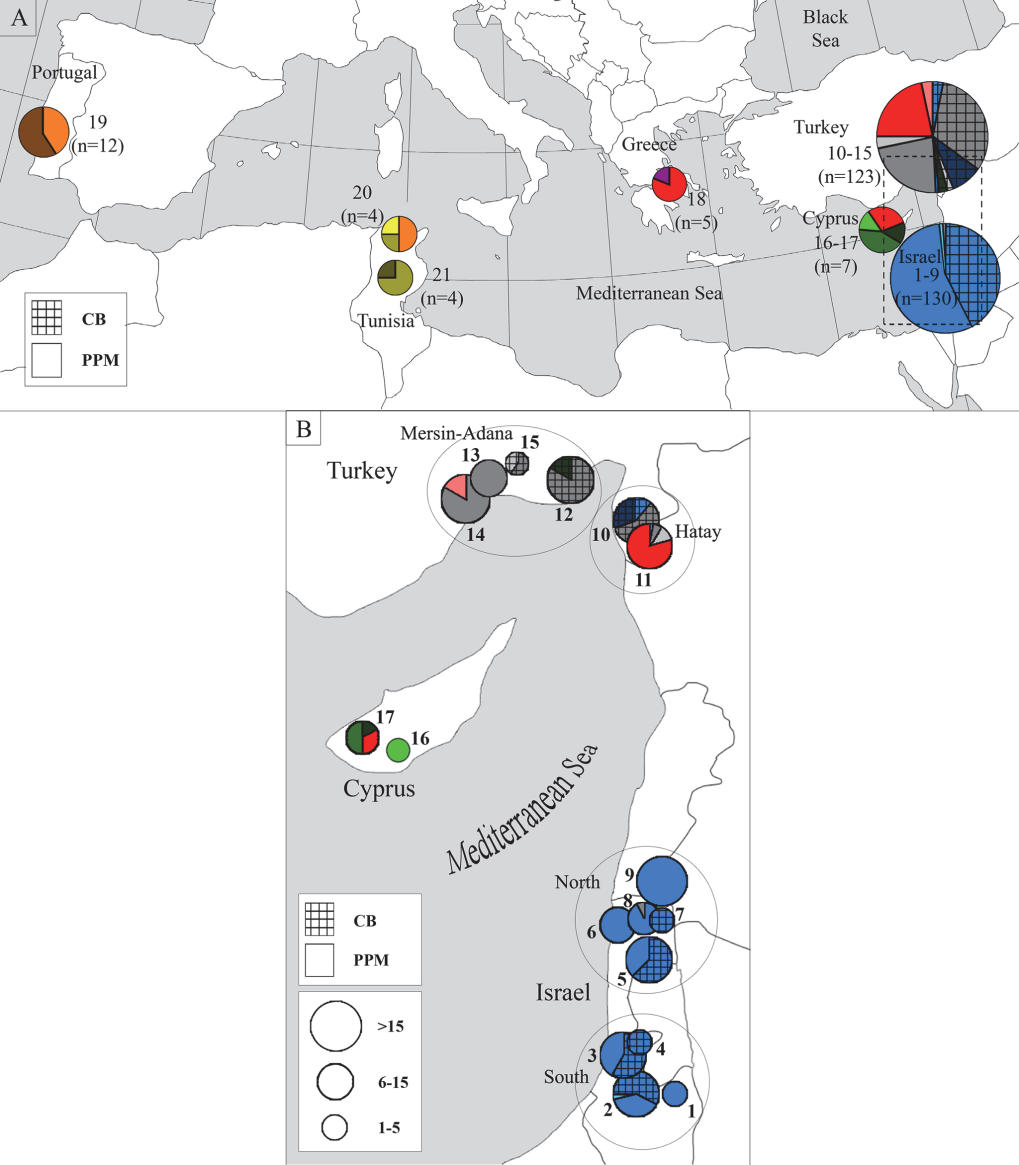
Geographical distribution of *Ooencyrtus pityocampae* COI haplotypes. 3A. Geographical mapping of Mediterranean OP haplotypes. Different colors represent different haplotypes, corresponding to the haplotype network shown in Fig 1. Haplotypes of Individuals taken from the caper bug eggs (= CB, in Turkey and Israel) are marked with cross lines. All other haplotypes belong to individuals that were sampled from the pine processionary moth (= PPM) populations. The numbers near each circle indicate the sample location codes provided in Table 1. The total number of individuals sampled in each area is indicated under the location codes. The area circled with dashed line is magnified in 3B. 3B. A detailed geographical mapping of haplotypes from Israel, Turkey and Cyprus. The size of the circles is proportional to the number of individuals sampled. The number near each circle represents the location code (see Table 1 for further data on samples from each location). The four main sampled areas in Israel and Turkey, e.g. Israel-south (locations 1–4), Israel-north (5–9), Turkey-Hatay (10–11) and Turkey-Mersin/Adana (12–15) are marked with black circles. This figure was modified from its original form and is similar but not identical to the original image, and thus is provided for illustrative purposes only.

A total of 16 different COI haplotypes were obtained (Table 2), comprising two to seven haplotypes from each sampled country; five in the West Mediterranean populations and 11 in the East Mediterranean populations, Fig 2). A maximum of five nucleotide substitutions differed within each pair of haplotypes, which corresponds to about 0.5% difference. Some of the haplotypes were shared among populations from different countries within each of the two main regions (East and West Mediterranean), but none were shared between the two regions (Fig 3A).

**Table 2:** Indices of genetic diversity for COI sequences of *Ooencyrtus pityocampae* populations from the various studied areas and hosts (CB = *Stenozygum coloratum*, PPM = *Thaumetopoea pityocampa/ T*. *wilkinsoni*).

Country	Region	Host	# sequences	Number of haplotypes	Haplotype diversity ***H*** (±SD)	Nucleotide diversity, π (±SD)	Өs (per sequence)*
Israel	South	CB	29	1	0.000±0.00	0.000±0.00	0.000±0.00
		PPM	24	2	0.083±0.07	0.000±0.00	0.268±0.07
	North	CB	26	1	0.000±0.00	0.000±0.00	0.000±0.00
		PPM	51	2	0.039±0.00	0.000±0.00	0.222±0.05
Turkey	Hatay	CB	36	3	0.570±0.06	0.001±0.00	0.482±0.13
		PPM	34	4	0.362±0.10	0.001±0.00	0.978±0.30
	Mersin-Adana	CB	23	3	0.379±0.12	0.000±0.00	0.542±0.16
		PPM	30	2	0.239±0.09	0.000±0.00	0.252±0.06
Cyprus		PPM	7	4	0.810±0.13	0.002±0.00	1.633±0.06
Greece		PPM	5	2	0.400±0.23	0.002±0.00	2.400±2.29
Portugal		PPM	12	2	0.530±0.00	0.001±0.00	0.662±0.25
Tunisia		PPM	8	4	0.750±0.13	0.001±0.00	1.540±0.92
**Total**			**285**	**16**	**0.710±0.02**	**0.001±0.00**	**2.408±0.60**

* *Өs* estimated population effective size multiplied by the mutation rate.

In spite of the large samples, very low genetic variability was detected among the Israeli populations (H = 0.000–0.083) (Table 2, Fig 3B). In fact, out of 130 individuals sampled from both hosts, only two were genetically different from the rest, and those by only a single nucleotide substitution. This low genetic diversity was found to be characteristic of the Israeli population; all other sampled populations appeared to be much more variable (H = 0.239–0.810), even populations from which only 10 or fewer samples could be collected, i.e., Greece, Cyprus and Tunisia.

Significant genetic differences (based both on Φ_st_ and Jost's D distances) were found between most populations, except among the Israeli populations, between Hatay-PPM and Greece, and between Greece and Cyprus (Table 3). However, these results might be influenced by the small sample size for the Greek and Cypriot populations (*n* = 5 and *n* = 7 respectively). In addition, Jost's D distances did not exclude the possibility of null distances between the Turkish populations, except for the Hatay-PPM population, which appeared to be significantly different from the other three tested populations.

**Table 3:** Pairwise divergence of *Ooencyrtus pityocampae* populations.

	Israel				Turkey				Cyprus	Greece	Portugal	Tunisia
	South		North		Hatay		Mersin-Adana					
	CB	PPM	CB	PPM	CB	PPM	CB	PPM	PPM	PPM	PPM	PPM
	1	2	3	4	5	6	7	8	9	10	11	12
1	-	0.000	0.000	0.000	**0.845**	**0.964**	**1.000**	**1.000**	**1.000**	**1.000**	**1.000**	**1.000**
2	0.008	-	0.003	0.000	**0.842**	**0.964**	**1.000**	**1.000**	**1.000**	**1.000**	**1.000**	**1.000**
3	0.000	0.000	-	0.000	**0.845**	**0.964**	**1.000**	**1.000**	**1.000**	**1.000**	**1.000**	**1.000**
4	0.000	0.009	0.000	-	**0.827**	**0.962**	**0.981**	**0.980**	**1.000**	**1.000**	**1.000**	**1.000**
5	**0.445**	**0.409**	**0.432**	**0.496**	-	**0.930**	0.131	0.151	**1.000**	**1.000**	**1.000**	**1.000**
6	**0.811**	**0.788**	**0.803**	**0.844**	**0.613**	**-**	**0.632**	**0.927**	0.452	0.000	**1.000**	**1.000**
7	**0.852**	**0.809**	**0.843**	**0.867**	**0.267**	**0.911**	**-**	0.018	**0.954**	**1.000**	**1.000**	**1.000**
8	**0.893**	**0.856**	**0.888**	**0.897**	**0.300**	**0.633**	**0.089**	**-**	**1.000**	**1.000**	**1.000**	**1.000**
9	**0.805**	**0.750**	**0.789**	**0.851**	**0.365**	**0.396**	**0.298**	**0.392**	**-**	0.422	**1.000**	**1.000**
10	**0.905**	**0.865**	**0.896**	**0.927**	**0.597**	0.012	**0.675**	**0.718**	0.232	**-**	**1.000**	**1.000**
11	**0.833**	**0.789**	**0.822**	**0.868**	**0.429**	**0.640**	**0.445**	**0.520**	**0.370**	**0.569**	**-**	**0.595**
12	**0.858**	**0.809**	**0.846**	**0.889**	**0.457**	**0.645**	**0.498**	**0.583**	**0.352**	**0.531**	**0.391**	**-**

Populations were divided according to area and host (CB = *Stenozygum coloratum*, PPM = *Thaumetopoea pityocampa/ T*. *wilkinsoni*). The *Φ*_*ST*_ and Jost's D values are shown below and above the diagonal, respectively; those that were significantly different from 0 (*P* < 0.05) are printed in **bold**).

The AMOVA analyses of the Turkish specimens did not reveal structures among regions or among hosts (Table 4). In both analyses, about half of the total genetic variation was attributed to variations among populations and the other half to variations within populations. The AMOVA analysis that partitioned the genetic diversity between West Mediterranean and East Mediterranean populations indicated that about 23.5% of the genetic variance was due to variations between regions, but this was not found to be significant (*p* = 0.13). Again, half of the genetic variance was attributed to variation among populations. The Mantel test, however, showed significant results: R = 0.239, *p* = 0.04 when *Φ*_*st*_ was used; R = 0.402, *p* < 0.0001 when Jost's D was used—indicating a correlation between geographic and genetic distances. It is worth noting that this result is still significant or marginally significant when the Israeli populations are excluded: R = 0.218, *p* = 0.09 when *Φ*_*st*_ is used; R = 0.450, *p* = 0.008 when Jost's D is used.

**Table 4:** Analysis of molecular variance for *Ooencyrtus pityocampae* populations considering different groupings of the sampled sites.

Grouping	Source of variation	d.f	Sum of squares	Variance components	Percentage of variation	Fixation Indices
**Turkish regions** (Adana-Mersin/Hatay)	Among groups	1	9.819	-0.06138 Va	-8.68	ΦCT: -0.08679
					
	Among populations within groups	2	27.102	0.43323 Vb	61.26	ΦSC: 0.56367*
					
	Within populations	119	39.908	0.33536 Vc	47.42	ΦST: 0.52580*
					
	Total	122	76.829	0.70721		
**Hosts** (*Stenozygum coloratum*/*Thaumetopoeae pityocampa*-*T*. *wilkinsoni*)	Among groups	1	15.586	0.07150 Va	9.51	ΦCT: 0.09513
					
	Among populations within groups	2	21.335	0.34474 Vb	45.87	ΦSC: 0.50690*
					
	Within populations	119	39.908	0.33536 Vc	44.62	ΦST: 0.55380*
					
	Total	122	76.829	0.7516		
**Main regions** (West Mediterranean/East Mediterranean)	Among groups	1	12.959	0.12233 Va	13.79	ΦCT: 0.13794
					
	Among populations within groups	7	107.101	0.53864 Vb	60.74	ΦSC: 0.70458*
					
	Within populations	276	62.333	0.22584 Vc	25.47	ΦST: 0.74533*
					
	Total	284	182.393	0.88681		

* = Significant differences (*P* < 0.05).

Tajima's D test supported the existence of non-random selection processes in the Israeli population (D = -1.34, *p* = 0.02), and the negative value may be an indication of a recent expansion (see discussion). In contrast, no such evidence was observed for the Turkish population (D = 0.28, *p* = 0.66).

### ITS2 analysis

The ITS2 sequencing revealed almost no detectable polymorphism between individuals. This lack of genetic differences in the ITS2 supported the assumption that the OP-PPM and OP-CB populations were identical, but made this DNA fragment unusable for the genetic distances. There was, however, a single and consistent difference that differentiated between the West and East

**Fig 4:**
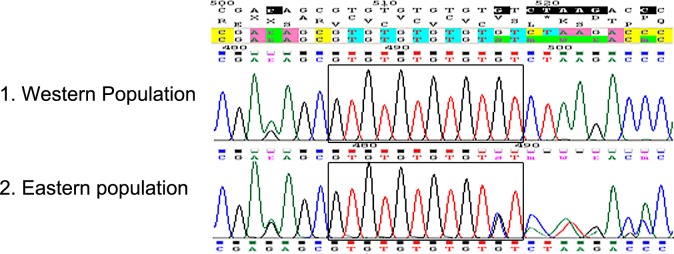
DNA chromatograms of a segment from the ribosomal ITS2 gene of *Ooencyrtus pityocampae*. This segment contains a microsatellite (starting from nucleotide 507) in which a variable number of GT repeats is present. Microsatellite repeats are framed with a black rectangle. 1. All individuals from the West Mediterranean population (Portugal and Tunisia) have a fixed number of six GT repeats. 2. All individuals from the East Mediterranean population (Israel, Turkey, Cyprus and Greece) have mixed fragments with variable number of five or six GT repeats. Sequences were aligned with ChromasPro ver. 1.6 [61].

Mediterranean populations. Each individual from the Eastern population had a variable number of GT repeats (five or six) in a specific microsatellite located at the middle of the ITS2 segment (starting from nucleotide 507). This caused variability in sequence length, which varied between 930 and 932 bp (Fig 4). As a result, the sequencing of the full ITS2 segment of individuals from the Eastern population could only be obtained by sequencing from both directions, i.e., with both the forward and reverse primers. In contrast, Individuals from the Western population showed no within-individual polymorphism in the number of GT repeats, which was fixed at six. Consequently, sequence length was always 932 bp and the sequence was fully readable from both directions.

### AFLP analysis of the Israeli OP population

**Fig 5:**
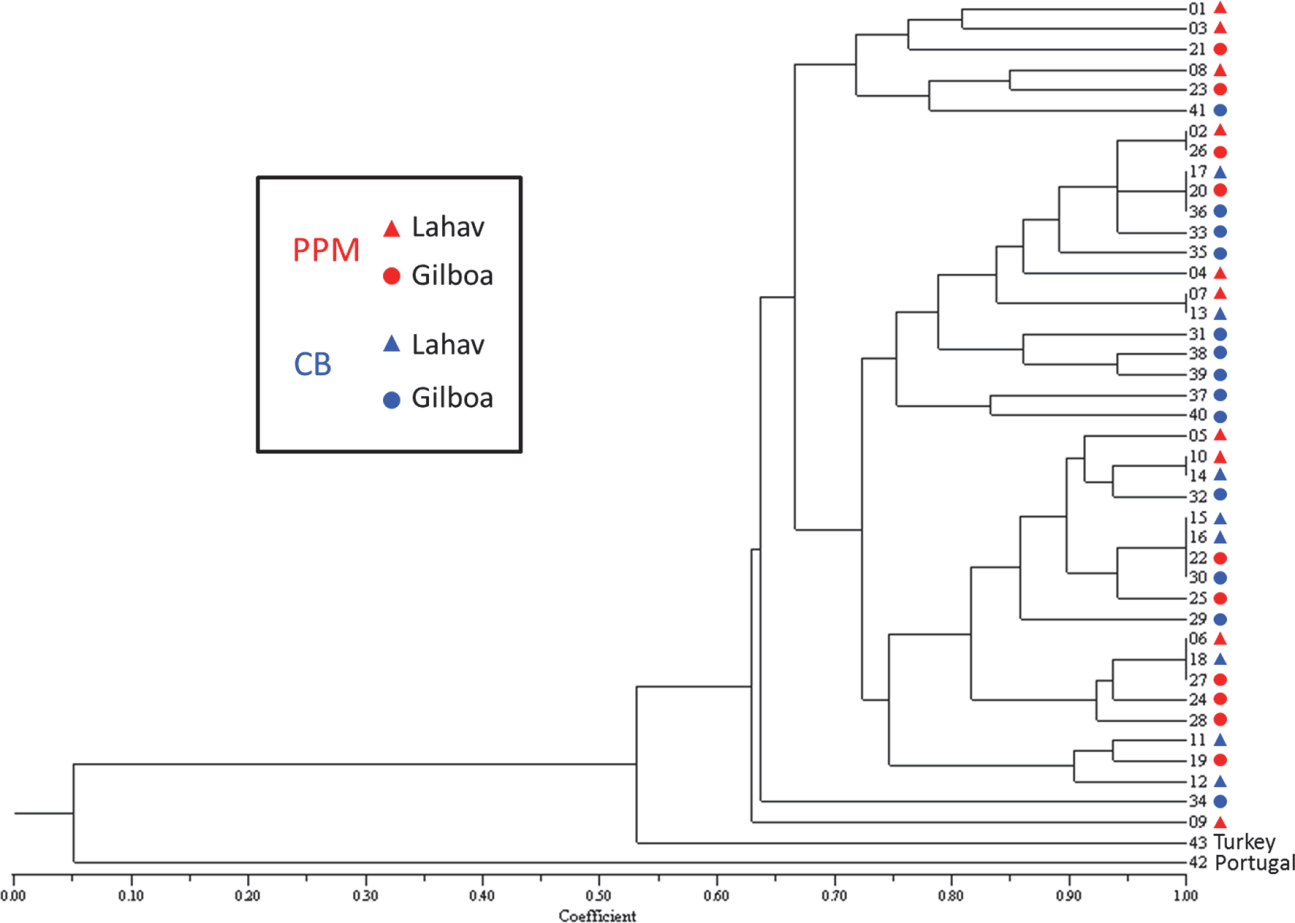
Dendrogram of *Ooencyrtus pityocampae* samples based on AFLP analysis. Data is based on the scores of 48 AFLP markers using the “Jaccard” similarity coefficient. Samples 1–41 belong to the Israeli population and were collected in Lahav (triangles) or Gilboa (circles) pine forests, from the eggs of the pine processionary moth, *Thaumemtopoea Wilkinsoni* (= PPM, marked in red), or the caper bug, *Stenozygum coloratum* (= CB, marked in blue). A single individual from CB in Turkey and a single one from PPM in Portugal were used as outgroups.

Since fingerprints for the two samples of *O*. *corei* did not share a sufficient number of fragments with the other samples, they were not included in the analysis. For PC E12/M49, no polymorphic markers were observed. Scoring the fingerprints generated with the remaining three PCs resulted in a data set of 48 polymorphic markers. The dendrograms based on the matrix generated with the use of the Simple matching, Jaccard and Dice coefficients had cophenetic correlations of 0.96, 0.94 and 0.97, respectively, which indicates that the dendrograms provide good representation of the similarities between the samples. It was observed that the individual from Portugal (sample 42) was the most distantly related to all other individuals (Fig 5). The other 42 samples (including a single individual from Turkey) showed more similarity. Also, on the basis of this data set, six small groups of individuals (two to four individuals per group) with identical marker scores were observed (Fig 5). Five out of the six groups included individuals from both hosts. These data further support the hypothesis that there is no difference between the OP-PPM and the OP-CB populations.

## Discussion

The major objective of the present study was to address the question of whether OP populations alternate seasonally between the two specific studied hosts, PPM and CB. This information has practical relevance for preservation and enhancement of OP populations. Since the eggs of each host are not found throughout all the OP activity period, the wasp population would have difficulty in surviving on either of the hosts alone, which apparently makes the alternation obligatory. Previous biological data supported the alternation hypothesis, i.e., the ability of OP individuals from either host to switch hosts in the laboratory [32]. Nevertheless, this ability does not prove that alternation also occurs in the field. It is worth noting that 13 other *Ooencyrtus* species are known to exploit both Lepidopteran and Heteropteran hosts [37]. However, there was no molecular analysis to support the morphological identification, or confirm the presence of each species in each of its apparent hosts. For this reason, and in light of the difficulty in identification of species in the genus, the true host ranges and the occurrence of host alternation in these species should remain questionable.

The molecular approach enabled us to avoid the difficulties of obtaining direct evidence for the occurrence of alternation in the field, because tracking the movement of such small insects between hosts is difficult and time consuming. Furthermore, the behavior of laboratory-reared individuals in field experiments may not necessarily reflect the typical behavior of naturally occurring field individuals.

Sequence data have shown that populations from both hosts are genetically similar, which supports the hypothesized occurrence of host alternation between PPM and CB. Even in light of the AFLP data, which were shown to better differentiate between individuals and populations in this species, there was no indication for host-related differentiation in the Israeli population. The same was found for the Turkish OP populations: four out of the seven COI haplotypes found in Turkey belonged to individuals from both hosts.

In general, most parasitoids are thought to have a rather specific niche, and most are confined to a specific group of host plants or host species [62]. Most examples of seasonal host alternation in this group involve aphid parasitoids (Hymenoptera: Aphidiidae). Some members of the subfamily attack several aphid species that are found at differing times of the year [63–65]; this behavior is thought to have major importance in the conservation of these parasitoid populations, and probably contributes to the biological control of some aphid pests. The situation in OP seemed similar, in that the two apparent hosts, the pine processionary moth and the caper bug, are found in different seasons. However, OP's case can be considered somewhat unique, in that not only are the two hosts found on distinct host plants (pine and caper), but they also belong to distinct taxonomic groups (Lepidoptera and Heteroptera).

Apart from the alternation issue, the present study also yielded detailed data on genetic structure and diversity of OP populations from several areas in the Mediterranean basin. The overall data revealed clear geographical-associated differences between OP populations, but no clear evidence for host-associated differences. There were substantial genetic differences between the Israeli and Turkish studied OP populations. The overall level of genetic diversity in the Israeli population was extremely low, although the AFLP data revealed some degree of genetic diversity, which was not evident in the apparently conserved COI and ITS2 sequences.

In Turkey the situation was somewhat different. In spite of geographical proximity and also similar sample sizes, the overall level of genetic diversity in all Turkish populations was much higher than that in the Israeli populations. Seven haplotypes were found in Turkey, compared with only three in Israel, of which two were each obtained from a single individual. *Φ*_*ST*_ and Jost's D distance methods showed that almost every one of the Turkish populations was genetically distant from the others (Table 3). Yet, this applied not only to populations from different areas, but also to those from different hosts within given sites. Apparently, because of the relatively high level of genetic diversity in the Turkish populations, a much larger sample size is required in order to obtain a satisfactory representation of the overall genetic picture, than is required for the Israeli populations. We suspect that this could also account for the observed differences in the frequency of the respective haplotypes among the various sampled populations from the different hosts and areas. The AMOVA analysis further confirmed that host based differences in the Turkish populations were not significant (Table 4). Despite the relatively small sample sizes, OP populations from other studied countries also showed a higher degree of polymorphism than the Israeli population, which confirms that the situation in Israel is unique. Interestingly, the overall picture resembles that of PPM, of which the Israeli populations were closely related to the populations from Southern Turkey, and showed lower levels of diversity than those from other studied areas [66,67].

Some available data on the occurrence of pine forests and the PPM in Israel may further help to account for the current genetic structure of OP. It is suggested that pine was very rare in cis-Jordan areas (Israel and the Palestinian territories) until recent centuries [68]. The PPM itself was discovered in the area only in 1937 in a small pocket of trees in Samaria [69]. Earlier surveys conducted in the region failed to find this species whereas other currently rarer *Thaumetopea* spp. were collected [70–72], suggesting that PPM was previously absent from the area, or existed in very small relict populations [73]. Most of the Israeli pine forests were planted only during the last 100 years or so, in the course of large afforestation projects [68]. However, small relict stands of Aleppo pine (*Pinus halepensis*) were most likely present much earlier, and it is possible that the PPM survived undetected in these small pockets of trees [74]. The fast spread in pine-planted area was accompanied by rapid buildup of the PPM population, and the Israeli pine area was completely covered by PPM by 2009–10 [75].

Interestingly, OP was the only *Ooencyrtus* species found on PPM eggs, and it is the dominant egg parasitoid on this host, whereas in other hosts' eggs, including those of the CB, OP is quite uncommon, probably as a result of extreme competition with other *Ooencyrtus* spp. In fact, OP was rarely found outside the PPM distribution range [36]. Furthermore, OP was never found on CB eggs collected in Israel's hottest and driest inland areas—the Negev Desert and the Jordan Valley—from which the PPM is absent, although in this case harsh climatic conditions may also account for its absence. These data suggest that OP is largely dependent on the PPM for its survivaland may even be unable to survive where this host is not found. Accordingly, the Israeli OP population, which did not previously exist, or may have survived as small numbers of individuals for thousands of years, dramatically increased in numbers following the rapid spread of the PPM in Israel since about 1940. The Tajima's D value of the Israeli population supports the rapid expansion hypothesis, and this may account for the low genetic diversity observed in this population.

The question of whether OP is dependent on PPM for its survival remains open. If OP population occurred in the area before the migration of PPM; its genetic diversity is expected to be similar to that of the Turkish population, unless dramatic bottleneck events had occurred in this area. The other possible scenario is that the occurrence of OP in Israel is a result of a founder effect associated with a relatively recent migration, following the arrival of PPM to Israel. According to Simonato [66], the Israeli and Lebanese PPM populations were diverged from the eastern Turkish population 0.22–1 Million years ago. If OP is dependent of PPM its arrival most likely occurred later at some point. A recent arrival following the expansion of the PPM range (founder effect), or occurrence for a much longer period, possibly with occasional bottleneck events [76], followed by a recent population expansion, could both account for the low genetic diversity found in the Israeli population.

A strong geographical/genetic structure was also observed in the populations around the Mediterranean, with the populations of the West Mediterranean having different mitochondrial COI haplotypes from those of the East Mediterranean. This separation was further emphasized by the small but consistent difference in the nuclear ITS2. A single individual from Portugal that was used in the AFLP analysis also showed a high level of divergence, both from the Israeli OP individuals and from the one from Turkey (Fig 5). Results of the present study generally reflect a similar picture to what was observed in the PPM genetic structure found by Kerdelhué et al. [67], who identified three main PPM clades as being geographically well structured: one that covered most of southwestern Europe and western North Africa; an eastern North African clade in Tunisia and adjacent countries; and a "wilkinsoni" clade in the Near East. The last is well separated from the first two clades. However, the overall level of genetic diversity in OP population was low when compared with that of the PPM, and some haplotypes were shared over rather large areas, e.g., populations of Turkey, Cyprus and Greece share haplotype 7, and those of Tunisia and Portugal share haplotype 13, whereas no such sharing was found among the PPM populations [67]. This is somewhat surprising since it can be assumed that the dispersal ability of PPM—males in particular [77,78]—is much higher than that of OP, in light of flight-distance data of other parasitoid species of approximately the same size [79,80]. The parthenogenetic reproductive system of OP may also account for its low genetic diversity [81,82], and the sharing of OP haplotypes between geographically distant populations may be the result of the overall low genetic diversity in this species. This low genetic diversity may be the result of a process of genetic sweep caused by an infection with an endosymbiont [83]; in the present case *Wolbachia*, which is known to be associated with OP [84], might be involved, and is probably also responsible for the parthenogenetic reproductive mode in this parasitoid.

In the present study we focused on the OP population of the East Mediterranean, a region where the geographical ranges of CB and PPM overlap. Drawing a complete picture of OP genetic structure throughout its area of distribution demands that more areas be surveyed. This is especially true for the West Mediterranean countries in southern Europe and North Africa, from which relatively small samples could be obtained in the present study. Preferably, further analysis should include multiple methods or additional gene segments, because the DNA fragments used in the present study, i.e., COI and ITS2, were shown to only partially distinguish between populations of this species from different locations and countries. Combining additional genetic information derived from AFLP, highly polymorphic microsatellites, or sequencing of additional gene fragments, would facilitate better differentiation between some geographically close populations, as was seen in the Italian OP populations [84] and in the Israeli population (present study). Furthermore, given the high level of morphological polymorphism in OP, and the fact that distinguishing individuals of this species from their congeners can be extremely difficult (J. Noyes, personal communication), it is suggested that future research that includes identification of OP individuals should also incorporate molecular analysis.

The scarcity of evidence for OP presence in areas from which PPM is absent, its rarity on other hosts, and, on the other hand, its emergence pattern in spring when PPM eggs are absent, raise fundamental questions for further research: (1) Is OP dependent on alternative hosts for survival until PPM eggs become available, or is its survival possible on PPM alone? (2) How important is the role of CB in particular, in OP conservation? (3) If OP cannot survive in areas where PPM is absent, should the designation of OP as "generalist" be revised?

Finally, the present study points to the importance of alternative hosts as a major tool in conservation biological control, and supports the idea that enrichment of pine forests with host plants for CB or other pentatomids is likely to lead to an increase in OP population. The existence of OP on CB and other pentatomids found on caper plants [47] supports the attribution of a generalist nature to this parasitoid. Identifying additional hosts may prove important for biological control efforts, because different summer hosts species are most likely present in different areas.
